# A 2‐year randomized blinded controlled trial of a conditionally licensed *Moraxella bovoculi* vaccine to aid in prevention of infectious bovine keratoconjunctivitis in Angus beef calves

**DOI:** 10.1111/jvim.15633

**Published:** 2019-10-12

**Authors:** Annette O'Connor, Vickie Cooper, Laura Censi, Ella Meyer, Mac Kneipp, Grant Dewell

**Affiliations:** ^1^ Department of Veterinary Diagnostic and Production Animal Medicine College of Veterinary Medicine, Iowa State University Ames Iowa; ^2^ The University of Sydney, Sydney School of Veterinary Science New South Wales Australia

**Keywords:** animal welfare, corneal scarification, infectious bovine keratoconjunctivitis

## Abstract

**Background:**

Infectious bovine keratoconjunctivitis (IBK) in beef cattle has major welfare and production implications. Effective vaccination against IBK would also reduce antibiotic use in beef production.

**Objective/Hypothesis:**

To evaluate the efficacy of a conditionally licensed commercial IBK vaccine containing *Moraxella bovoculi* bacterin. Primary working hypothesis was that animals vaccinated with 2 doses of the commercial *M. bovoculi* vaccine would have a lower risk of disease.

**Animals:**

Spring born calves at a university cow‐calf herd. After excluding animals with ocular lesions, calves eligible for prevention assessment in 2017 and 2018 were 163 (81 vaccinated, 82 unvaccinated) and 207 (105 vaccinated, 102 unvaccinated). One hundred sixty two and two hundred and six calves completed the follow‐up period in 2017 and 2018, respectively.

**Methods:**

A randomized controlled trial. The trial design was a 2‐arm parallel trial with a 1:1 allocation ratio.

**Results:**

In both years, calves receiving the vaccine had more IBK. This effect was small. The pooled risk ratio was 1.30 (95% confidence interval 0.84–2.01). The pooled unadjusted difference in mean weight (kg) at weaning was −0.88 (95% confidence interval—7.2‐5.43).

**Conclusions and Clinical Importance:**

We were unable to document that the *M. bovoculi* bacterin vaccine had a protective effect for the incidence of IBK in our single herd in a 2‐year study.

## 1 INTRODUCTION

Infectious bovine keratoconjunctivitis (IBK) is a common ocular infection of beef cattle, particularly in calves. Clinical signs of IBK range in severity from a mild conjunctivitis to severe ulceration and corneal perforation. Recovery is common in most animals; however, permanent blindness and corneal scarring can occur. IBK is not only a welfare concern, but also an economic one causing decreased weight gain.^1^

The main causative agent for IBK is *Moraxella bovis* for which there are commercial bacterins available. Challenge studies show that introduction of *M. bovis* leads to IBK.^2^, ^3^ *Moraxella bovoculi* was identified in 2007 in IBK cases and is a putative causal organism^4^, ^5^ Despite being over 10 years since the first report, the causal role of *M. bovoculi* remains unclear.^1^, ^6^, ^7^ In 2017, the first USDA conditionally licensed product for the prevention of IBK using *M. bovoculi* as the antigen base was marketed. The publicly available data to support the conditional licenses is based on product safety, that is, no adverse reactions, rather than prevention of IBK lesions. No publicly available data about prevention of IBK using this product are available. As a consequence, we conducted a trial to evaluate if the vaccine would prevent naturally occurring IBK in a herd that consistently presents with IBK.

The efficacy of pinkeye vaccines and treatments should be tested against naturally occurring disease. Experimental challenge studies might be too contrived to resemble the real‐world situations.^8^ To prove IBK causation by *M. bovis*, researchers reporting in the peer reviewed literature have challenged by unnatural routes,^9^, ^10^ used large doses of highly virulent inoculum,^11^, ^12^ limited the range of cofactors^11^, ^13^ and preconditioned corneas.^2^, ^12^, ^14^, ^15^, ^16^, ^17^, ^18^, ^19^, ^20^, ^21^ In contrast, when testing efficacy of Moraxella‐derived vaccines challenge culture was dropped onto the corneal surface^22^ or instilled into the conjunctival sac and applied it to palpebral surfaces by cotton‐tipped swab without any preconditioning.^23^, ^24^ For vaccines licensed for use in the United States by the Department of Agriculture's Center for Veterinary Biologics, it is not possible to know the approaches to challenge as these are not publicly available.

The objective of this study was to evaluate the association between vaccination for *M. bovoculi* and the cumulative incidence of IBK and weight gain in beef calves. Our primary working hypothesis was that, if effective, animals vaccinated with 2 doses of the conditional licensed commercial *M. bovoculi* vaccine (USDA CVM code: 2A77.00, Addison Biological Laboratory, Inc # 355) would have a lower risk of disease. We tested this hypothesis by assessing the cumulative disease risk from enrollment to weaning in vaccinated and unvaccinated animals. A secondary working hypothesis was related to weight gain. As diagnosis of IBK can be imperfect on pasture‐based animals and IBK is strongly associated with decreased weight gain, our working hypothesis was that if vaccination is effective, then the mean weaning weight of animals vaccinated with conditional licensed commercial *M. bovoculi* vaccine (USDA CVM code: 2A77.00, Addison Biological Laboratory, Inc # 355) would be higher when compared to animals that did not receive vaccine.

## 2 MATERIALS AND METHODS

### 2.1 Ethics statement and registration

The Animal Care and Use Committee of Iowa State University reviewed and approved the study protocol in 2017 and 2018. The protocol for the study conducted in 2018 was preregistered and is available online at the Open Science Framework (doi: 10.17605/OSF.IO/ZJ4WM). No major deviations from the approved protocol were made during the study. The reason for not preregistering the study in 2017 is that the senior author was unaware of the availability of this option.

### 2.2 Owners and animals

Calves used in the study were owned by Iowa State University McNay Research and Demonstration Farm. This farm was selected because it is convenient to the study staff; we have a history of working with the farm, and it has a consistent incidence of IBK.^25^, ^26^, ^27^, ^28^, ^29^ The farm is located in Lucas County, Iowa, and houses approximately 260 spring‐born (February to May) Angus calves each year. IBK has been a long‐standing problem in this herd with >15% of calves affected each season.^27^, ^30^ Because it is unlikely that other causes of ocular lesions would result in disease in >15% of the herd, the definition of an IBK‐affected herd was used in the absence of other definitions.

The farm separates the calves into the management groups defined by the predominating dam parity. These are not strictly enforced: mostly cows 2–3 years old, 4–7 years old, more than 7 years old. In 2017, calves from the 2‐year‐old cows in the 2‐ to 3‐year‐old management group were not eligible to participate in this study because this group was enrolled in another project. In 2018, all groups were eligible for enrollment. In 2017 and 2018, all eligible calves for enrollment were spring‐born and were >2 months of age with no visible ocular lesions or scars. This eligibility was assessed without knowledge of assignment. Any ocular lesion including tearing, blepharospasm, conjunctivitis, or corneal lesions was reason for exclusion.

The McNay Farm has been used for prior IBK vaccine trials^25^, ^27^, ^30^ and a population‐based cohort study^28^ because of the history of IBK occurrence in the herd. The presence of *M. bovoculi* and *M. bovis* in the herd has been documented by several previous studies.^27^, ^30^, ^31^

### 2.3 Interventions

Calves received either:

- Two 2 mL doses of the commercially available *M. bovoculi* vaccine (https://addisonlabs.com/product/moraxella-bovoculi-bacterin/) administered SC (https://www.bqa.org).
- No treatment in 2017 and in 2018; two 2‐mL doses of saline administered SC.

In the 1st year, the group decided not to include an extra injection (ie, placebo) as we considered it unnecessary. In the 2nd year, we still considered a saline injection unnecessary; however, during the protocol review stage, a reviewer requested inclusion of a saline placebo and we did not consider it to be problematic to make this change. Our group routinely views placebo and saline as exchangeable nonactive controls and are unaware of evidence to suggest otherwise. An adjuvant only placebo would not be considered exchangeable. The study used naturally occurring IBK; therefore, no challenge model details are required.

### 2.4 Objectives

The objective was to determine if the *M. bovoculi* vaccine could control IBK. The primary goal was to estimate the effect of vaccination with the conditional *M. bovoculi* vaccine on the cumulative incidence of IBK. The effect estimate used is the risk ratio. The secondary goal was to estimate the effect of vaccination with the conditional *M. bovoculi* vaccine on mean weight of the vaccinated versus unvaccinated group. The effect estimate was difference in mean weaning weight at the end of the trial.

### 2.5 Outcomes

The primary outcome was the occurrence of IBK, which was defined as any ocular lesion, tearing, blepharospasm, conjunctivitis, corneal lesions, or corneal scars consistent with IBK. Farm staff were trained by the research staff to diagnose IBK. This training occurred during enrollment sessions in this and previous years. The research and the field staff groups evaluated eyes and discussed the definition of IBK each year at enrollment and outcome assessment on the occasions when they were present. At weaning, if calves were observed with centrally located corneal opacity (ie, corneal scars) that was considered consistent with a healed IBK lesion, and they had not previously been diagnosed with IBK, they were considered to have IBK. It was not necessary for staff to observe a centrally located IBK ulcer to diagnose animals with IBK as it was important to treat animals as soon as they were diagnosed for welfare reasons. Given the consistent occurrence of IBK in this herd, this is a pragmatic solution to manage cattle on pasture. Farm staff were unaware of the vaccination status of the calves. It was considered that any over‐diagnosis would be equivalent across groups. The secondary outcome was weight at weaning.

### 2.6 Sample size

The expected sample size was calculated based on the expected cumulative incidence previously reported at McNay Farm (>30%).^7^ Using 3 randomized blinded trials from a systemic review of IBK vaccine efficacy, the risk ratio of 0.5 was used as the vaccine efficacy.^6^ The calculation parameters were α value of .05, power of 80% and 2‐sided test with assumed independence of all enrolled units. The resulting sample size calculation using these parameters suggested that 120 calves were required per treatment group. As it was known in 2017, that that only 180 calves would be eligible, a 2‐year study was planned. All spring‐born calves were enrolled, which was approximately 270 animals in 2018. This enrollment meant that the study was able to detect a smaller difference in IBK incidence than originally planned because of the larger sample size. The sample size was calculated in Open Epi software.^32^

### 2.7 Randomization: Sequence generation

The trial design was a 2‐arm parallel trial with a 1:1 allocation ratio. The randomization schedule was created by an investigator (AOC) using the statistical software, R package for designing and analyzing randomized experiments and R Foundation for Statistical Computing.^33^ A chute processing order sheet was created using the randomized schedule. Allocation was at the individual animal level.

### 2.8 Randomization: Allocation concealment

At the first vaccination dose allocation, calves entered the chute and were assessed for eligibility, that is, absence of ocular lesions, by the farm staff without knowledge of the allocation. If no lesions were observed, the calf was eligible to be enrolled for the first dose.

### 2.9 Randomization: Implementation

Once a calf was deemed eligible by the blinded farm staff, the research staff administered either the first dose of the vaccine or the placebo (nothing in 2017 and a saline injection in 2018) based on the pregenerated allocation schedule. If an animal was ineligible at 1st enrollment, this was recorded, and no further action was taken. For the 2nd dose of the vaccine, the animal was identified by the farm staff and the eyes evaluated for ocular lesions again. If ocular lesions were present, this information was recorded and the animal was not considered eligible for subsequent analysis but still received the allocated treatment (vaccinated or unvaccinated). The rationale for administering the 2nd dose of the allocated treatment to animals with ocular lesions was that, if the vaccine was ultimately found to be effective, then these ineligible animals might still benefit from the 2nd vaccination dose. This was based on the desire to maximize the potential animal welfare benefits to the animals.

### 2.10 Blinding (masking)

Blinding of outcome assessment was achieved by ensuring that the animal ID tags did not indicate the treatment. For an animal's vaccination status to be known at the time of IBK diagnosis, it would require that the McNay staff committed the allocation received at enrollment to memory. Such knowledge is unlikely for all animals but possible for some. The allocation sheet was also not stored on the farm so it could not be “looked up” later. The primary outcome of interest is the treatment for IBK based on the presence of clinical signs associated with IBK such as corneal opacity, corneal ulcer, corneal perforation, or blepharospasm with tearing and conjunctivitis, or a scar associated with IBK. At weaning, we collected information about the presence of corneal scars (centrally placed corneal opacity) and active lesions. Again, the allocation status was not known unless the farm staff had committed the allocation from 4 months before memory, which seems unlikely. The calf weaning weight was collected during normal processing. The approach to weighing is unlikely to be biased by knowledge of the treatment received, given the pace of work and other processing requirements. Before analysis, without knowledge of the allocation status, the weight data were assessed for unusual observations to be excluded, that is, abnormal weights. The association between the outcome and the allocation status was assessed after data cleaning.

### 2.11 Statistical methods

Only animals that completed enrollment, that is, no ocular lesions at the 1st or 2nd vaccination, were included in the primary analysis. The primary analysis for the impact of vaccination on the cumulative incidence used a Mantel–Haenszel analysis stratified by year. Assessment of effect modification by year was conducted by using the Breslow and Day chi‐square test for effect modification by year. The effect measure reported was the Mantel–Haenszel risk ratio and 95% confidence interval in each study year and pooled across the years. The numerator for all risk ratio calculations was the incidence of IBK in vaccinated animals and the denominator was the incidence in unvaccinated animals. If vaccination was associated with reduced incidence of IBK, the risk ratio would be <1.

The secondary analysis for weaning weight used a linear model to assess if there was effect modification by year using a cross product term between year and treatment, that is, did the effect of the vaccine differ by year. A *P* value <.1 was considered significant. The effect measure was mean difference in weaning weight between the vaccinated and unvaccinated cattle. If there was no evidence of effect modification, then a pooled mean weaning weight was calculated. We subtracted the mean weight of the unvaccinated calves from the vaccinated calves (mean weight in vaccinated calves—mean weight in unvaccinated calves). Therefore, if the vaccinated gained more weight, this mean difference would be positive, that is, on average more weight gained by the vaccinated animals.

Several unplanned ancillary unplanned analyses were conducted. We calculated the risk ratio for IBK for calves that were born before April 1 in either year. This was done because, although we vaccinated all animals for pragmatic disease‐control reasons, the manufacturer's label suggests calves should be 14 weeks of age at vaccination. We calculated the mean difference and 95% confidence interval in weaning weights of vaccinated compared to unvaccinated animals, adjusted for weight at enrollment, study year and sex. The mean difference and 95% confidence interval in weaning weights of IBK positive compared to IBK negative animals adjusted for study year was also calculated. For all other analyses, we did not use significance testing for the vaccine effect instead preferring to report the effect measure (risk ratio or mean difference) and precision of the effect measure (95% confidence interval).^34^, ^35^, ^36^ All analyses were conducted in R software.^37^

## 3 RESULTS

### 3.1 Study flow

The flow of animals born at the McNay herd in 2017 and 2018 is reported in Table 1. The number of animals eligible at the first vaccination in 2017 and 2018 was 183 and 268, respectively. The reason for the difference in enrollment numbers is that in 2017, calves born to young cows were participating in another study. In 2017, between 1st and 2nd vaccine doses, a larger number of animals developed IBK in the vaccinated group than the unvaccinated group (13 versus 7). In 2018, a similar number of calves developed lesions between doses (30 vaccinated versus 31 unvaccinated).

**Table 1:** Flow of study participants through the study and results for the primary outcome (infectious bovine keratoconjunctivitis incidence) and the secondary outcome (weaning weight) for beef calves enrolled in a 2‐year randomized controlled trial of a conditionally licensed commercial *Moraxella bovoculi* bacterin in the United States

Characteristics	2017		2018	
Born at farm	277		286	
Exclusion reason preenrollment				
Enrolled in another project	75		NA	
Died before enrollment	19		18	
Ocular lesion at 1st vaccination	0		0	
Allocated at 1st vaccination	183		268	

	Vaccinated	Placebo	Vaccinated	Placebo
Allocated at 1st dose: n_1_	94	89	135	133
Mean summer weight kg (SD)	148 (27)	142 (29)	102 (24)	105 (24)
Female (% of n_1_)	49 (52%)	39 (44%)	73 (54%)	57 (43%)
Ocular lesion at 2nd dose (% of n_1_)	13 (14)	7 (8)	30 (22)	31 (23)
Completed 2nd enrollment n_2_ (% of n_1_)	81 (86%)	82 (92%)	105 (78%)	102 (77%)
Mean enrollment weight kg (SD)	150 (28)	143 (30)	102 (24)	108 (21)
Missing enrollment weight	1	1	0	0
Female (% of n_2_)	44 (54%)	36 (44%)	55 (53%)	43 (42%)
Died (% of n_2_)	0	1	1	0
Complete data analyzed for IBK: n_3_	81	81	104	102
IBK diagnosed (% of n_3_)	22 (27%)	17 (21%)	16 (15%)	12 (12%)
Mean weaning weight (SEM)	220 (3.18)	210 (3.69)	209 (3.07)	218 (2.90)
Missing weaning weight	0	0	0	1
Calves born before April 1 (n_4_)	54	43	67	76
IBK diagnosed (% of n_4_)	19 (35%)	13 (30%)	24 (36%)	22 (28%)

### 3.2 Recruitment

The dates of recruitment for 1st and 2nd dose of the vaccination and placebo are described in Table 2. Although the calves were scheduled to receive the 2nd dose of the vaccine between 21 and 28 days after the first dose, this was often not possible. In a decision beyond the control of research staff, the farm staff canceled working on the calves because of welfare concerns in extreme heat during both summers. In those circumstances, the animals received the 2nd dose at the next practical opportunity based on weather and farm staff availability. In 2017 and 2018, the calves were weaned between the 1st and 3rd weeks of October.

**Table 2:** The dates of vaccination for calves enrolled in 2‐year randomized controlled trial of conditionally licensed commercial *Moraxella bovoculi* bacterin trial in 2017 and 2018 in beef calves in the United States

	2017			2018		
Management Group	1st dose	2nd dose	Days in between	1st dose	2nd dose	Days in between
Two‐ and 3‐year‐old dams	Not enrolled			May 31, 2018	June 22, 2018	22
Old (>7 y) dams	June 19, 2017	July 21, 2017	32^a^	June 11, 2018	July 9, 2018	28
Young (4‐7 y) dams	June 7, 2017	June 26, 2017	19	June 13, 2018	July 19, 2018	36^a^

a Group was vaccinated outside the 21‐ to 28‐day recommended range because of rescheduling necessitated by excessive heat considered to create welfare concerns for calves.

### 3.3 Baseline

The characteristics of the calves eligible for the study, that completed enrollment, and that were included in the analysis are presented in Table 1. Differences in baseline characteristics at enrollment were not tested for significance as per recommendations.^38^ As some animals did develop IBK lesions between the 1st and 2nd dose of vaccine, we also report the baseline of the animals that completed the 2 doses. In 2017, more calves in the vaccinated group (13 of 94, 14% of those initially allocated) developed IBK between the 1st and 2nd dose than the placebo group (7 of 89, 7% of those initially allocated).

### 3.4 Numbers analyzed

One animal was lost to follow‐up because of death between completion of enrollment and completion of the study (Table 1). For the IBK outcome, data were available for all calves. One vaccinated and 1 placebo calf were missing an enrollment weight in 2017 and none in 2018. For the weaning weight data, 1 calf in the placebo group had an illegible weight record on the data sheet in 2018, so this animal was included in the IBK data analysis but not the weaning weight data analysis.

### 3.5 Outcomes and estimation

**Figure 1:**
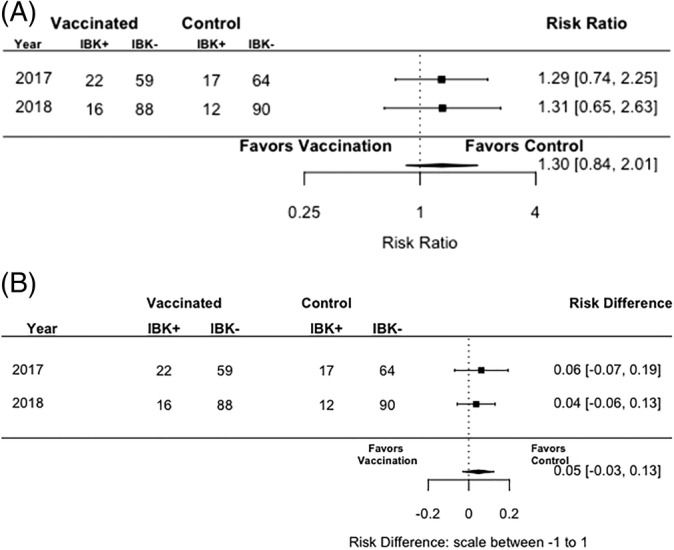
Unadjusted risk ratio (A) and unadjusted risk difference (B) for the effect of vaccination with 2 doses of a commercial *Moraxella bovoculi* vaccine or a saline control injection on the incidence of infectious bovine keratoconjunctivitis in Angus calves in 2017 and 2018

The results for the primary outcome, cumulative incidence of IBK in both groups each year is reported in Table 1 and the pooled risk ratio was 1.30 with a 95% confidence interval from 0.84, to 2.01(Figure 1). The test for heterogeneity over years was not significant (*P* > .1), which is obvious when evaluating the risk ratio in 2017 and 2018, as both are similar. The results suggest that vaccinated calves had increased risk of IBK in both years, although the uncertainty around the estimate suggests that no effect of the vaccine is also possible. The difference in risk of disease between the vaccinated and unvaccinated animals is only 5% as shown by the risk difference scale (see Figure 1).

The results for the secondary outcome, weaning weight in vaccinated and unvaccinated calves are reported in Table 1. The preplanned unadjusted analysis of vaccine effect on weaning weights is presented in the top panel of Figure 1.

There was no evidence of an effect of the vaccine status on the mean weaning weight. There was a significant effect of year on the effect of vaccination of weaning weight (interaction *P* = .03). The difference in enrollment weights of the vaccinated and placebo calves that completed the enrollment is only slightly different from those initially allocated suggesting that randomization was effective for this variable (Table 1). Each year more females were randomly allocated to the vaccinated group (Table 1). We present the original planned unadjusted estimation approach in Table 3 for each year and combined years.

**Table 3:** The estimate of effect of vaccination with conditionally licensed commercial *Moraxella bovoculi* vaccine on weaning weight of calves

Model	Covariates included in model	Vaccination effect size (kg)
2017 only	Vaccination	9.78 (0.25‐19.3)
2018 only	Vaccination	−9.32 (−17.6 to −1.02)
Combined model 1	Vaccination	−0.88 (−7.2 to 5.43)
Combined model 2	Vaccination, year	−0.88 (−7.2 to 5.46)^a^
Combined model 3	Vaccination, year, sex	0.63 (−5.6 to 6.8)^a^
Combined model 4	Vaccination, year, sex, enrolment weight	0.16 (−3.34 3.6)^a^

*Note*: The vaccination effect size is the mean difference in weaning weight in kg (mean weaning weight in kg in vaccinated group compared—mean weaning weight in kg in unvaccinated group).

a Adjusted for covariates in the model.

### 3.6 Ancillary analyses

The pooled risk ratio of IBK incidence for calves born before April 1 was 1.27 (95% CI–0.8‐1.7), which is not very different from the analysis including all ages (Table 1). Three unplanned adjusted estimation approaches with different covariates are included Table 3. It can be seen that the effect size for the vaccination status is consistently close to the null value of zero regardless of covariates included. The ancillary analysis of the effect of IBK on weaning weight showed that calves with an IBK diagnosis weighed on average less than calves not diagnosed with IBK in the follow‐up period (mean difference = −10.541 kg, SE 4.14, 95% confidence interval—18.6 to −2.4).

### 3.7 Adverse events

No adverse reactions to the vaccination were reported by the farm staff.

## 4 DISCUSSION

### 4.1 Interpretation

The results of this 2‐year study suggest that the USDA Animal and Plant Health Inspection Service (APHIS) licensed commercially available *M. bovoculi* vaccine was not associated with reduced incidence of IBK in a herd that has a consistently high incidence of IBK.

The vaccine was used in 2 conditions. In 2017, IBK lesions had begun to occur before the full vaccination schedule was completed; however, the majority of cases (39 of 59 cases) occurred after the vaccination process was completed. The overall prevalence of IBK in the herd in 2017 was 32% (59/183). In 2018, the circumstances differed, with the majority of cases (61/89, 70%) occurring between the 1st and 2nd vaccination, although the overall prevalence of IBK was also 33% (89)/(135 + 133). These changing circumstances are associated with the nature of field trials and the realities of naturally occurring IBK. Interestingly, the effect of the vaccine was not impacted by these differences. In both years, calves eligible for prevention assessment had a slightly higher risk of IBK than their unvaccinated herd mates. This effect was very small, as can be seen in risk difference in Figure 1.

We did not attempt to culture the organism from the eyes of calves. Historically, in this herd, *M. bovis* and *M. bovoculi* have been recovered or identified via culture or PCR methods from active IBK lesions suggesting that both organisms are present in the herd.^7^, ^26^, ^27^, ^39^ However, our group has argued against the idea that recovery of an organism from an active lesion as an approach to assessing the causal organism. Because eye swab specimens cannot be acquired immediately after initiation of infection and, owing to the potential for opportunistic secondary infection, it is not possible to make causal inference about organisms isolated from active lesions. As a result, we decided the best way to diagnose IBK in the calf herd was not on the basis of organisms recovered from old lesions but rather the clinical signs consistent with IBK, coupled with the long history of IBK in the herd and high infectivity rate.

The vaccine manufacturer's labeled protocol recommends that calves should be 14 weeks of age when vaccinated and receive 2 doses with a 3‐ to 4‐week interval. In our herd, such an approach is not feasible because most IBK occurs in June–July–August and therefore some calves were not 14 weeks when vaccinated; furthermore, inclement weather prevented repeat vaccination within 21 days. However, we did evaluate if the vaccine effect using different age criteria and found no meaningful difference in the effect.

Randomized trials should be assessed for risk of bias in 5 domains. For bias caused by allocation approach for this item, this study should have a low risk of bias, as we concealed allocation until after eligibility was assessed and used a random number generator to allocate animals to treatment. Consequently, baseline differences are caused by random chance. We have presented adjusted and unadjusted estimates for end users to consider. For a bias caused by deviations from intended interventions, this would require differential caregiving of animals based on vaccination status. We consider this as unlikely (low risk of bias) as the farm staff were, for the most part, unable to differentially care for animals, as groups are commingled on pastures. The risk of bias because of differential outcome assessment is also unlikely as farm staff were unable to determine the vaccine status of the calf when evaluating the occurrence of IBK or a corneal scarring. Another concern related to measurement might be bias toward the null value of the risk ratio (ie, 1) because of nondifferential measurement of the outcome. Staff might over diagnose IBK, because the field‐based nature of the study means that research staff are not in daily attendance. Such over diagnosis would be nondifferential, that is, occur equally in both groups; it might lead to a dilution of the presence of IBK, thus reducing the power to detect a difference. As the ancillary analysis documents that calves diagnosed with IBK have sustainably lower weaning weights than calves not diagnosed with IBK, this finding argues against a large impact of possible misdiagnosis on the vaccination comparison. If a large number of calves diagnosed with IBK did not have IBK, then the difference in weaning weight would also have been diluted.

Finally, bias because of selective reporting is not possible as we have reported the primary and secondary outcome of interest and identified when the analysis is ancillary as documented in the prepublished protocol.

The vaccine is registered with a conditional license, and therefore explicit data of efficacy are not available. This implies that USDA AHPIS Center for Veterinary Biologics (CVB) had made the determination that there is reasonable expectation of efficacy for the biologic agent (the *M. bovoculi* antigen[s] in this case) against pinkeye hence the label claim “for the vaccination of healthy cattle 14 weeks of age or older against pinkeye (infectious bovine keratoconjunctivitis).” At the time of publication, information informing the “reasonable expectation*”* was not publicly available. These data are the first publicly available assessment of the efficacy of this product.

### 4.2 Generalizability

As with all studies, each yearly trial was a single random event and it is essential that replicated results are available to determine if the findings are consistent with other studies. The conclusion of CVB to provide a conditional license would imply that the results are not consistent with other evidence. If we consider this publicly available body of work in the larger body of work about *M. bovis* or *M. bovoculi* bacteria's role in IBK, the results are consistent with other findings from our group about vaccines targeting IBK, that is, no evidence of a protective effect.^2^, ^7^, ^25^, ^27^ Further, it is possible that earlier timing of the vaccine relative to pinkeye occurrence, with boosters given at least 2 weeks before the onset of pinkeye season might have led to different results. In our herd, this is not feasible, but it might be in other herds.

Across the entire body of work this might be considered robust because this consistency occurs in considerable heterogeneity of years, ages at vaccination, and timing of disease pressure albeit on 1 farm. If just seeking to make inferences about this product, then the degree of uncertainty is high as it is for any product only assessed in 2 trials. The potential mechanisms of why vaccines are not protective include the targeted organism not being causal, the immunologic response not being protective, or the difficulty of inducing a protective response for bacteria. The results of this study provide no insights as to the mechanism of vaccine failure. Finally, we might speculate that had this trial indicated the product was effective, the results could still be considered more convincing. Single studies that demonstrate a protective effect for vaccination appear to be treated with less skepticism than single studies that do not demonstrate an effect.

### 4.3 Overall evidence

We evaluated a commercial *M. bovoculi* vaccine and where unable to document that it was associated with reduced disease incidence or increased weight gain.

#### Conflict of Interest Declaration

Annette O'Connor serves as Associate Editor for the *Journal of Veterinary Internal Medicine*. She was not involved in review of this manuscript.

#### Off‐label Antimicrobial Declaration

Authors declare no off‐label use of antimicrobials.

#### Institutional Animal Care and Use Committee (IACUC) or Other Approval Declaration

Iowa State University IACUC approval, 18‐095.

#### Human Ethics Approval Declaration

Authors declare human ethics approval was not needed for this study.

## References

[1] O'Connor AM , Shen H , Wang C . Descriptive epidemiology of *Moraxella bovis*, *Moraxella bovoculi* and *Moraxella ovis* in beef calves with naturally occurring infectious bovine keratoconjunctivitis (pinkeye). Vet Microbiol. 2012;155:374‐380. 21962410 10.1016/j.vetmic.2011.09.011

[2] Gould S , Dewell R , Tofflemire K , et al. Randomized blinded challenge study to assess association between *Moraxella bovoculi* and infectious bovine Keratoconjunctivitis in dairy calves. Vet Microbiol. 2013;164:108‐115. 23452750 10.1016/j.vetmic.2013.01.038

[3] Rosenbusch RF , Ostle AG . *Mycoplasma bovoculi* infection increases ocular colonization by *Moraxella ovis* in calves. Am J Vet. Res. 1986;47:1214‐1216. 3729120

[4] Angelos JA , Ball LM . Hess JF. Identification and Characterization of Complete RTX Operons in Moraxella Bovoculi and Moraxella Ovis Veterinary Microbiology. 2007;125:73‐79. 17561358 10.1016/j.vetmic.2007.05.009

[5] Angelos JA , Spinks PQ , Ball LM , et al. *Moraxella bovoculi* sp. nov., isolated from calves with infectious bovine keratoconjunctivitis. Int J Syst Evol Microbiol. 2007;57:789‐795. 17392208 10.1099/ijs.0.64333-0

[6] Burns MJ , O'Connor A . Assessment of methodological quality and sources of variation in the magnitude of vaccine efficacy: a systematic review of studies from 1960 to 2005 reporting immunization with *Moraxella bovis* vaccines in young cattle. Vaccine. 2008;26:144‐152. 18054816 10.1016/j.vaccine.2007.10.014

[7] O'Connor A , Brace S , Gould S , et al. A randomized clinical trial evaluating a farm‐of‐origin autogenous *M. bovis* vaccine to control infectious bovine keratoconjunctivitis in beef cattle. J Vet Intern Med. 2011;25:1447‐1453. 22092641 10.1111/j.1939-1676.2011.00803.x

[8] Doohoo I , Martin W , Stryhn H . Veterinary Epidemiological Research. Charlottetown, Prince Edward Island, Canada: VER Inc.; 2009.

[9] Chandler RL , Turfrey BA , Smith K . Development of a laboratory animal model for infectious bovine keratoconjunctivitis. Res Vet Sci. 1982;32:128‐130. 7045996

[10] Poels J . Keratitis infectiosa der Runderen (keratitis polybacillosa). Tijdschr Veeartsenijk. 1911;38:758‐766.

[11] Rogers D , Cheville N , Pugh G Jr . Pathogenesis of corneal lesions caused by *Moraxella bovis* in gnotobiotic calves. Vet Pathol. 1987;24:287‐295. 3617395 10.1177/030098588702400401

[12] Ruehl WW , Marks CF , Fernandez R , et al. Purification, characterization, and pathogenicity of *Moraxella bovis* pili. J Exp Med. 1988;168:983‐1002. 2902184 10.1084/jem.168.3.983 PMC2189028

[13] Chandler RL , Turfrey BA , Smith K , et al. Virulence of *Moraxella bovis* in gnotobiotic calves. Vet Rec. 1980;106:364‐365. 7376396 10.1136/vr.106.16.364

[14] Gerber JD , Frank SK . Enhancement of *Moraxella bovis* induced keratitis of mice by exposure of the eye to ultraviolet radiation and ragweed extract. Am J Vet Res. 1983;44:1382‐1384. 6881676

[15] George LW . Clinical infectious bovine Keraotconjunctivitis. Comp Cont Edu. 1984;6:712‐722.

[16] Lepper AWD , Power BE . Infectivity and virulence of Australian strains of *Moraxella bovis* for the murine and bovine eye in relation to pilus serogroup sub‐unit size and degree of piliation. Aust Vet J. 1988;65:305‐309. 2904256 10.1111/j.1751-0813.1988.tb14512.x

[17] Ryoo J‐P . Hydrogel ocular inserts for the treatment of infectious bovine keratoconjunctivitis Biomedical Engineering. Ames, Iowa: Iowa State University; 1989:115.

[18] Hughes DE , Pugh GW . Ultraviolet radiation and *M. bovis* in the etiology of infectious bovine keratoconjunctivitis. Am J Vet Res. 1965;26:1331‐1338. 5882548

[19] Hughes DE , Pugh GWJ , McDonald TJ . Experimental bovine infectious Keratoconjunctivitis caused by sunlamp irradiation and *Moraxella bovis* infection: determination of optimal irradiation. Am J Vet Res. 1968;29:821‐827. 5642355

[20] Pugh GWJ , Hughes DE . Infectious bovine keratoconjunctivitis induced by different experimental methods. Cornell Vet. 1971;61:23. 4925115

[21] Jackson FC . Infectious keratoconjunctivitis of cattle. Am J Vet Res. 1953;14:19‐25. 13030997

[22] Smith P , Blakenship T . Effectiveness of two commercial infectious bovine keratoconjunctivitis vaccines. Am J Vet Res. 1990;51:1147‐1150. 2389894

[23] Lepper A , Elleman TC , Hoyne PA , et al. A *Moraxella bovis* pili vaccine produced by recombinant DNA technology for the prevention of infectious bovine keratoconjunctivitis. Vet Microbiol. 1993;36:175‐183. 7901935 10.1016/0378-1135(93)90138-w

[24] Billson F , Tennent J , Lepper A , et al. Protective efficacy of a *Moraxella bovis* haemolytic fraction in a challenge trial. Aust Microbiol. 1993;14:A‐34.

[25] Cullen JN , Engelken TJ , Cooper V , et al. Randomized blinded controlled trial to assess the association between a commercial vaccine against *Moraxella bovis* and the cumulative incidence of infectious bovine keratoconjunctivitis in beef calves. J Am Vet Med Assoc. 2017;251:345‐351. 28703672 10.2460/javma.251.3.345

[26] Cullen JN , Lithio A , Seetharam AS , et al. Microbial community sequencing analysis of the calf eye microbiota and relationship to infectious bovine keratoconjunctivitis. Vet Microbiol. 2017;207:267‐279. 28757034 10.1016/j.vetmic.2017.07.003

[27] Funk L , O'Connor A , Maroney M . A randomized and blinded field trial to assess the efficacy of an autogenous vaccine to prevent naturally occurring infectious bovine kerato‐conjunctivitis (IBK) in beef calves. Vaccine. 2009;27:4585‐4590. 19531391 10.1016/j.vaccine.2009.05.082

[28] Funk L , Reecy J , Wang C . Associations between infectious bovine keratoconjunctivitis at weaning and ultrasonographically measured body composition traits in yearling cattle. J Am Vet Med Assoc. 2014;244:100‐106. 24344858 10.2460/javma.244.1.100

[29] O'Connor AM , Brace S , Gould S , et al. A randomized clinical trial evaluating a farm‐of‐origin autogenous *Moraxella bovis* vaccine to control infectious bovine keratoconjunctivis (pinkeye) in beef cattle. J Vet Intern Med. 2011;25:1447‐1453. 22092641 10.1111/j.1939-1676.2011.00803.x

[30] O'Connor AM , Brace S , Gould S . A randomized clinical trial evaluating a farm‐of‐origin autogenous *Moraxella bovis* vaccine to control infectious bovine keratoconjunctivitis (pinkeye) in beef cattle. J Vet Intern Med. 2011;25:1447‐1453. 22092641 10.1111/j.1939-1676.2011.00803.x

[31] Shen H , Gould S , Kinyon J . Development and evaluation of a multiplex real‐time PCR assay for the detection and differentiation of *Moraxella bovis*, *Moraxella bovoculi* and *Moraxella ovis* in pure culture isolates and lacrimal swabs collected from conventionally raised cattle. J Appl Microbiol. 2011;111:1037‐1043. 21827584 10.1111/j.1365-2672.2011.05123.x

[32] Dean AG , Sullivan KM , Soe MM . OpenEpi: Open Source Epidemiologic Statistics for Public Health, 2013. www.OpenEpi.com. Accessed June 2019.

[33] R Development Core Team . R: A Language and Environment for Statistical Computing. Vienna, Austria: R Foundation for Statistical Computing; 2018.

[34] Kenneth R , Sander G , Timothy L . Modern Epidemiology. 3rd ed. Philadelphia: Wolters Kluwer Health/Lippincott Williams & Wilkins; 2008.

[35] International Committee of Medical Journal Editors . Preparing for Submission. Available from: http://www.icmje.org/recommendations/browse/manuscript‐preparation/preparing‐for‐submission.html. Accessed June 2019.

[36] Wasserstein RL , Lazar NA . The ASA's statement on p‐values: context, process, and purpose. Am Stat. 2016;70:129‐131.

[37] R Core Team . R: A language and environment for statistical computing. Vienna, Austria: R Foundation for Statistical Computing; 2019 https://www.R-project.org/.

[38] de Boer MR , Waterlander WE , Kuijper LD , et al. Testing for baseline differences in randomized controlled trials: an unhealthy research behavior that is hard to eradicate. Int J Behav Nutr Phys Act. 2015;12:4. 25616598 10.1186/s12966-015-0162-z PMC4310023

[39] O'Connor AM , Shen HG , Wang C , et al. Descriptive epidemiology of *Moraxella bovis*, *Moraxella bovoculi* and *Moraxella ovis* in beef calves with naturally occurring infectious keratoconjunctivitis (pinkeye). Vet Microbiol. 2012;155:374‐380. 21962410 10.1016/j.vetmic.2011.09.011

